# Potato Late Blight Outbreak: A Study on Advanced Classification Models Based on Meteorological Data

**DOI:** 10.3390/s24237864

**Published:** 2024-12-09

**Authors:** Parama Bagchi, Barbara Sawicka, Zoran Stamenkovic, Dušan Marković, Debotosh Bhattacharjee

**Affiliations:** 1 Department of CSE, RCC Institute of Information Technology, Beliaghata, Kolkata 700015, India; paramabagchi@gmail.com; 2 Department of Plant Production Technology and Commodity Science, University of Life Sciences in Lublin, 20-950 Lublin, Poland; barbara.sawicka@up.lublin.pl; 3 Institute of Computer Science, University of Potsdam, An der Bahn 2, 14476 Potsdam, Germany; 4 IHP, Leibniz-Institut für Innovative Mikroelektronik, 15236 Frankfurt, Germany; 5 Faculty of Agronomy in Čačak, University of Kragujevac, 32000 Čačak, Serbia; dusan.markovic@kg.ac.rs; 6 Department of CSE, Jadavpur University, Kolkata 700032, India; debotosh@ieee.org

**Keywords:** potato late blight, machine learning, stacking classifier, logistic regression, prediction models, crop health management, meteorological data, agricultural forecasting, plant pathology

## Abstract

While past research has emphasized the importance of late blight infection detection and classification, anticipating the potato late blight infection is crucial from the economic point of view as it helps to significantly reduce the production cost. Furthermore, it is necessary to minimize the exposure of potatoes to harmful chemicals and pesticides due to their potential adverse effects on the human immune system. Our work is based on the precise classification of late blight infections in potatoes in European countries using real-time data from 1980 to 2000. To predict the potato late blight outbreak, we incorporated several hybrid machine learning models, as well as a unique combination of stacking classifier and logistic regression, achieving the highest prediction accuracy of 87.22%. Further enhancements of these models and the use of new data sources may lead to a higher late blight prediction accuracy and, consequently, a higher efficiency in managing potatoes’ health.

## 1. Introduction

In recent years, agricultural manifestation using machine learning techniques has increased. Late blight infection is a severe disease which has been affecting the growth rate of potatoes, mainly in the European countries. Late blight infection of potatoes is caused by the fungus termed as *P. infestans* and is a significant disease that could lead to the huge destruction of crops if proper control measures are not taken. The yield of potatoes could decrease tremendously to 80% in the forthcoming years.

The initial symptoms of this disease are visible, with small spots which appear circular and are light to dark green in color. This disease is so dangerous that they might cause a total decay in the tubers. Beyond that, the quality and productivity of the crops also diminish when *P. infestans* attacks the crops.

### 1.1. Background

Potatoes are a common source of carbohydrates consumed by people worldwide and imported by many other countries from Europe. Although the consumption of tubers infected with late blight, caused by *P. infestans* Mont De Bary, does not pose a direct threat to human health according to the latest research [1], this pathogen can attack plants, causing the tubers to rot and die, without producing toxins that would be harmful to humans. Eating infected tubers may reduce their nutritional value and affect their taste and smell. In addition, infected tubers may be more susceptible to infection by other microorganisms, mainly bacteria, which can produce harmful toxins. Therefore, although the tubers infected with late blight are not directly dangerous, it is recommended to avoid eating them and to implement the appropriate crop management to minimize the risk of secondary infections and huge economic losses [2]. Recently, late blight management strategies have been relying primarily on fungicide applications. However, their uncontrolled use poses a serious threat to the environment and human health. It also increases pathogen resistance and negatively impacts beneficial organisms like nitrogen-fixing bacteria, local antagonistic microorganisms, and mycorrhizal fungi. To minimize the use of fungicides, safe methods of managing potato late blight should be used, including paying attention to any early warning signs against this pathogen. In recent years, significant changes have been recorded in the isolates of the fungus causing late blight (types A1 and A2), and thus there have been changes in their aggressiveness towards potato and tomato crops. A strategy to control potato blight is to prevent *P. infestans* from affecting the potato crops [1,2]. Early detection of *P. infestans* should reduce the use of pesticides rather than fertilizers. This means that if *P. infestans* is detected early, before an outbreak, then we would not need the application of pesticides, which are primarily used to eliminate pests. Instead, we can apply fertilizers to promote the overall growth of the plants.

**Figure 1:**
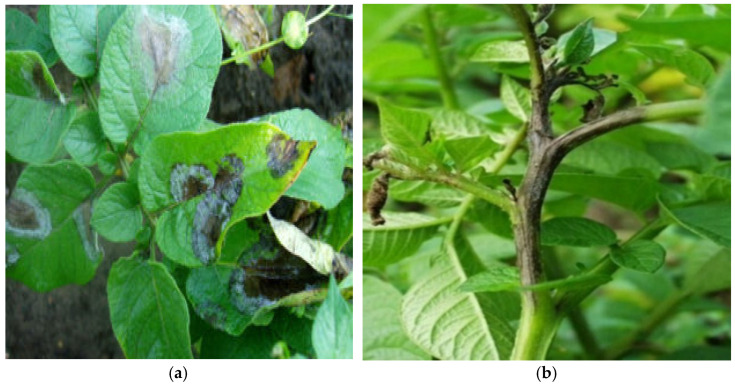

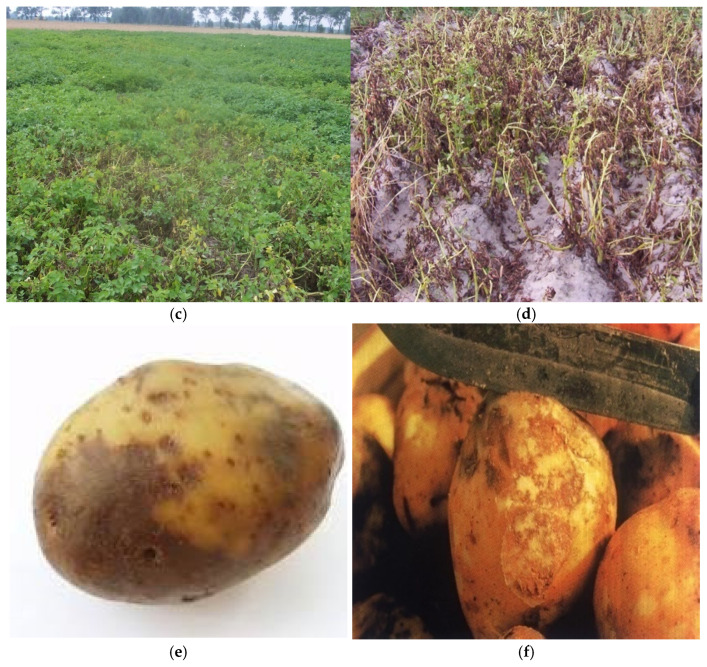
Potato late blight: (**a**) Leaf form *P. infestans*; (**b**) Stem form of potato blight on late cv. ‘Amarant’; (**c**) Potato infection with late blight in ‘Boryna’ cv.; (**d**) *P. infestans* plantation infection, 2°, scale 9°, ‘Irga’ cv.; (**e**) Potato late blight on the tuber; (**f**) Potato blight on the cross-section of tubers; Source: own.

Recent approaches to the ecological management of late blight have been increasingly using mathematical modeling and machine learning techniques to predict and monitor the spread of the disease. These models can analyze weather data, soil moisture, and other environmental factors to predict a potential potato blight outbreak. Thanks to early warning systems, farmers can take preventive actions at an early stage, minimizing the need to use fungicides. The use of such models enables precise resource management and a more targeted use of plant protection products, which is beneficial not only for the environment but also for the economics of agriculture. Moreover, the development of biotechnological technologies, such as gene editing using the CRISPR method, enables the creation of potato varieties with increased resistance to *P. infestans*, which also contributes to reducing the dependence on chemical plant protection products. Therefore, the integration of advanced modeling methods with traditional cultural practices and modern biological technologies provides a comprehensive approach to the ecological management of late blight, ensuring efficiency and sustainability in crop protection [1,2,3]. Figure 1a–f show the massive damage caused when *P. infestans* affects the crops.

### 1.2. Significance of the Presented Work

Disease management through cultural practices is the first line of defense, while a forecasting system, physiological strategies, biological control, host plant resistance, and biotechnological approaches are essential for the efficient, effective, and ecological management of late blight.

So, the significance of the present work is that we have devised several hybrid machine learning models to predict potato late blight infection much before it occurs. In this manner, we provide a robust way to combat this infection using the unified blend of stacking classifier with logistic regression which has resulted in 87.22% accuracy.

Most of the models used here to predict potato late blight infection are based on classification techniques because regression is used to predict the value of the infection based on the meteorological data, while classification is used to predict the categorical class labels of new instances based on the past observations. Here, we are mainly concentrating on the latter, because our labels are the target values used to predict the level of the infection, i.e., [1,2,3,4,5,6,7,8,9]. This infection level is the target variable with categorical values. Our feature values are the entire set of meteorological data. We felt that this problem could better be solved using a fusion of various classifiers. This is because we need to predict the level of infection. If the level of infection is predicted correctly well beforehand, then the farmers can take precautions so that late potato blight never occurs. This is exactly where classification is needed.

The outcomes indicate the importance of using advanced prediction models to foresee potato late blight. This allows growers to take proactive measures and prevent the disease instead of resorting to expensive fertilizers if the blight affects the crops, which can also pose risks to human health.

## 2. Related Work

Potatoes are an essential crop, but they are affected by pests worldwide. The most severe infection that tends to diminish the yield of potato blight is caused by *P. infestans*. This fungus causes a massive decrease in yield, and under the suitable atmospheric conditions, it can multiply at an alarming rate and destroy crops in a short time. Control is possible only through the use of fungicides. The survey conducted comprises two sections; we first discuss the studies conducted on late blight potato infections based only on the meteorological conditions and then on the use of a machine learning model for the prediction of potato late blight infections.

### 2.1. Survey on Potato Late Blight Infection

Bairwa et al. [3] studied potato late blight infections which caused 30–50% loss in kharif potato production. They conducted a survey on how to combat this dreadful infection and found that a (T3) prophylactic spray with mancozeb, followed by Sectin (fenamidon + mancozeb) after 7–10 days of the first and third sprays, as well as mancozeb again after the second spray, could help to lower the spread by 21.3% and maximize the yield.

González-Jiménez et al. [4] devised a model to control the losses caused by *P. infestans* in the aspects of disease growth rate, infection time, and temperature under climate change. They named the model POTATOPEST. The simulation results on this model depicted that the simulated actual yields which were obtained under the presence of *Phytopthora infestans* correlated well (R2 = 0.66) with the actual field conditions under meteorological variables. The work discussed how the impact of the disease, as well as the time when the infection had occurred, had affected the yield losses.

Zhu et al. [5] had devised three resistance genes, *Rpi-sto1*, *Rpi-vnt1.1*, and *Rpi-blb3*, against the *P. infestans* infection. The authors initially combined the three genes into one feature vector termed as pBINPLUS. The method showed a new way of stacking genes to provide strong resistance against *P. infestans*. Finally, it was proven that the use of *Avr* genes which matched the *Rpi* genes was effective in treating potato blight disease.

Wang et al. [6] studied the primary causes for the spread of *P. infestans* in Central Mexico. The study mainly focused on the spread in the areas of Central Mexico, along with a few areas from Michoacán, Tlaxcala, and Toluca. Based on the study conducted, the authors finally concluded that potato blight infection exists in Central Mexico, and this was a cause for the spread of the disease in the United States.

Duan et al. [7] investigated 189 different potato genotypes comprising 20 species from the *Solanum tuberosum* Andigenum and Chilotanum groups. They identified ten related sources and 127 stand-alone sources infected with *P. infestans*. Their results indicated that there was a huge diversity in the growth of potatoes and cultivated *S. tuberosum* Chilotanum. In conclusion, it was proven that there exists abundant genetic variation in both wild and cultivated potato germplasms, while the cultivated *S. tuberosum* Chilotanum group unequivocally exhibits lower genetic diversity.

Tsedaley et al. [8] discussed the biological, economic, and managerial impacts of late blight infection. The work described here specified that *P. infestans* has two phases, namely the survival and infection phases. The authors also discussed about the pathogen, cultural control, chemical control, and the life cycle of *P. infestans*. The final conclusion was that by adopting integrated disease management, *P. infestans* can be controlled.

Duan et al. [9] studied the resistance of 03112-233 towards the gene 90128 and found that it was based on a new “R” gene. To understand the susceptibility in 03112-233, a profiling analysis based on RNAseq was performed. A hierarchical clustering analysis was also carried out, and it was proven that different genes can depict different infection variants at different times.

Vleeshouwers et al. [10] observed that potato blight infections secrete AVR proteins, which are targeted by resistance proteins from the *Solanum* species. They investigated that most *Solanum* species traces are found in *P. infestans*, which exists in Central Mexico. The authors discussed, in detail, the resistance and variabilities of *P. infestans*. They concluded that engineering the “R” genes for the recognition of the pathogen was slowly becoming popular, necessary to develop a resistance against *P. infestans* infection.

Alvarez-Morezuelas et al. [11] studied 52 kinds of *P. infestans* from eight different regions in Spain. For this purpose, 17 different physiological races were found. They found that the most complicated race was “Cadiz”, which included 11 virulent factors from *Av1* to *Av11*. In this study, the authors studied the many different variants existing in Canada, the United States, and the United Kingdom, and then concluded that huge variants of *P. infestans* existed.

Amin et al. [12] studied three exotic varieties of potatoes, namely BARI Alu-90, BARI Alu-91, and BARI Alu-77, in Northern Bangladesh and studied the effect of *P. infestans*. Their study showed that BARI Alu-90 had the highest level of resistance compared to the other two varieties. In addition, the results proved that BARI Alu-90 and BARI Alu-91 were highly resistant to *P. infestans* and that BARI Alu-77 was moderately resistant.

### 2.2. Survey on Prediction of Potato Blight Infection Using ML Models

In this section, we discuss the approaches based on simulation models and decision support systems used to predict potato late blight infection.

The groundbreaking simulation model developed in 1981 by Fry et al. [13] paved the way for further advancements in the field. Their second model revolutionized our understanding of fungicide deposition and redistribution on potato leaves. Additionally, a forecasting model was established to consider the intricate relationships among weather, fungicide distribution, plant resistance, and the development of *P. infestans*. This led to the development of a simulation model that critically evaluated the importance of various plant resistance components.

The LATEBLIGHT model [14], developed in 2005, simulates the impact of weather, plant growth, resistance, and fungicide use on the development of *P. infestans*. In 2008, the LB2004 model was created and validated in various regions.

Decision support systems increase the effectiveness of potato late blight control strategies. The BLITECAST forecasting model was first developed in 1975. PhytoPRE was a computer-based decision support system in Switzerland, encompassing an epidemiological forecasting model and a set of decision rules. The PROGEB model included the PHYTEB component, which forecasted *P. infestans* and simulated the successive stages of infection. The SIMBLIGHT1 model required data on temperature, relative humidity, soil moisture, crop prevalence, and cultivar susceptibility [15]. Subsequently, in 2008, an online expert system was developed for potato growers in Michigan [16]. Small et al. [17] developed the BLIGHTPRO decision support system. INDO-BLIGHTCAST was created for managing potato late blight in India [18].

Recently, several machine learning models have been developed to predict late blight infections.

Vaidheki et al. [19] devised statistical models, such as autoregressive integrated moving average (ARIMA) and autoregressive integrated moving average with exogenous variables (ARIMAX), with other machine learning models, including neural network auto regression (NNAR), support vector regression (SVR), and regression tree (CART), to predict late blight in the northern part of West Bengal. The models were developed during the 3- and 7-day intervals using different meteorological conditions. The results indicated that CART was the best fitted model.

Meno et al. [20] monitored five crops in Northwest Spain. They concluded that mild temperatures and high humidity were factors which helped in the spread of aerobiological risk level of *P. infestans*. Also, the other factors which aggravated the disease were infection pressure, wind, and leaf wetness. Machine learning algorithms, random forest (RF) and C5.0 decision tree (C5.0), were used here, and they resulted in accuracies of 87% and 85%, respectively.

Kilonzi et al. [21] worked in the field where the fungicides were applied in alternations of one, two, or three applications at intervals of 7, 14, and 21 days. The concentrations of 70% and 100% of fungicides curtailed mycelial and blight lesion growth, while their 35% concentration reduced mycelial growth and lesion size by 53% and 2%, respectively. However, spraying the fungicides at weekly and bi-weekly intervals gave the highest yields of tubers compared to tri-weekly and unprotected plots. The authors observed that the maximum benefit was observed when the crops were protected using three fungicides applications, followed by two fungicide applications biweekly.

Fraiwan et al. [22] targeted the treatment of three kinds of corn diseases (namely Cercospora leaf spot, common rust, and northern leaf blight), in addition to healthy plants. The authors worked with 3852 leaf images, applied ten different CNN models, and obtained an accuracy of 98.6%.

None of the abovementioned methods forecasted the potato blight before its emergence. The climate, meteorological, and soil conditions must be considered to obtain such a prediction. These factors were missing in the above works. Without them, the growth of potato blight infection cannot be accurately predicted. It is also necessary to develop a hybrid system involving potato blight images and meteorological data for a more precise prediction, which will be a part of our future work. In the present study, machine learning is utilized to forecast the onset of potato late blight before its emergence.

The main objectives of the present work are as follows:

- A real-life dataset containing meteorological data spanning twenty years from 1980 to 2000 is introduced.
- This dataset is a valuable resource for researchers interested in studying and analyzing meteorological patterns and trends over an extended period, because it contains eighteen different features related to windspeed, temperature, humidity, etc., which will be essential for predicting the emergence of late potato blight.

So, the main aim of this study is to predict potato blight infection well before it occurs. Based on this, an alternative research hypothesis is outlined, which will allow agriculturists to efficiently reduce the occurrence probability of potato blight infection. Consequently, the development of various machine learning models aims to determine the most effective approach to anticipate the outbreak of potato blight infection in European countries, ultimately increasing crop yield and reducing the cost of pesticides.

## 3. Materials and Methods

We have collected, analyzed, and classified data on the potato late blight in Poland during the years 1983–1985 and 1987–1989, as well as long-term meteorological data during the years 1980–2000.

### 3.1. Dataset Description

The research utilized a dataset spanning 21 years (1980–2000) on *P. infestans* and continuous meteorological data from the potato growing season (April–September). The focus was on predicting the infection of potato leaves by *P. infestans* through the following two experiments:

- A fertilizer experiment;
- An experiment with the herbicide Sencor 70 WP on 44 potato varieties.

Field experiments were conducted in Jabłoń, Biala Podlaska district, on soil classified as a good rye complex, employing the randomized sub-blocks method in a dependent split-plot design, with three repetitions.

The main objectives included determining the optimal herbicide application timings as follows:

- Before emergence: Sencor 75 WP at 1 kg ha^−1^;
- After emergence: When shoots reached 10–15 cm, Sencor 75 WP at 0.75 kg ha^−1^;
- Standard practice: Afalon 50 WP at 2 kg ha^−1^.

Measurements included air temperature, ground temperature, air humidity, and wind speed, all of which were captured in 18 feature values per observation using a thermograph. The dataset, which included 44 potato varieties from all maturity groups, followed standard agricultural practices and assessed plant infection every seven days using a 9-point scale by Pietkiewicz et al. [23]. The plot size for harvest was 20 m², with standard practices for pre-crop and tuber planting, including rolling, harrowing, and earthling for herbicide application.

### 3.2. Experimental Settings

Agricultural practices were standardized from the previous crop’s harvest to the planting date of tubers. During the growing season, rolling and harrowing were performed post-shooting for after-emergence herbicide application. All experimental variants included the last plant earthing up and spraying with herbicides. No other agricultural practices were performed afterward. Mineral fertilization involved 90 kg N, 90 kg P_2_O_5_, and 135 kg K_2_O per hectare, with organic fertilization at 25 t ha^−1^. In vitro fungicide testing was conducted by adding herbicides Afalon 50 WP and Sencor 70 WP to a rye medium, inoculated with *P. infestans*. Inoculation involved transferring 7-day colonies grown on rye medium to herbicide-infused Petri dishes. Four repetitions were carried out for each herbicide concentration. Control dishes contained rye medium without herbicides. Incubation occurred at 20 °C, with colony growth observed after 4 and 8 days. Fungicidal activity was measured by spawn growth inhibition percentage using the Kowalik and Krechniak method [24].

In vitro investigations involved testing fungicides under laboratory conditions using the dish technique. Herbicides Afalon 50 WP and Sencor 70 WP were added to a rye medium inoculated with *P. infestans*. Concentrations were 0.5% for Afalon 50 WP and 0.2% and 0.25% for Sencor 70 WP. Fungicidal activity was measured by the percentage of spawn growth inhibition. These studies were conducted at the Department of Phytopathology, University of Agriculture in Lublin. Data included records of potato blight infection from the aforementioned years [23].

### 3.3. Climatic Conditions

The climatic conditions in Jabłoń, located in the Lublin district of Poland, are characterized by temperatures ranging from 2 °C to 20 °C, with ground temperatures between 8 °C and 20 °C. The geographical coordinates are 51°34′ latitude and 23°02′ longitude, at an altitude of 155 m above sea level. This region, like the rest of Central and Eastern Poland, experiences a moderate, transitional climate with high variability on both an annual and multi-annual scale, significantly impacting potato growing conditions and the risk of diseases such as potato late blight.

Average annual temperatures in this part of Poland range from 7 °C to 9 °C. Summers are moderately warm, with average temperatures in July of around 17 °C to 19 °C, while winters are mild, with average January temperatures of between −3 °C and −1 °C. Annual rainfall ranges from 500 to 700 mm, with the majority occurring in summer, particularly in July and August, where monthly rainfall can reach approximately 80–100 mm. High air humidity during the potato growing season (May–September) is typical for this region.

Sunshine duration is approximately 1600–1800 h per year, with around 450–500 h during the potato growing period, which is sufficient for proper plant growth. The average wind speed in this region is about 2–4 m/s, providing adequate ventilation for the plants and helping to reduce moisture on leaf surfaces. However, wind speeds can vary seasonally, with higher speeds of 5–6 m/s in winter and early spring, as well as lower speeds in summer.

In terms of temperature variation across Poland, studies have indicated an annual temperature increase at a rate of 0.2 °C per decade. The country has experienced several droughts, with durations ranging from 200 to 300 days, most of which were mild, although 5.2% to 10.7% of these droughts were more severe.

Extreme meteorological conditions, including droughts in 1982 and 2000, particularly affected Central and Southeastern Poland. These conditions likely influenced the rates of potato blight infection. By combining continuous observations of potato blight from 1983 to 1985 and 1987 to 1989 with long-term meteorological data from 1980 to 2000, we evaluated the dataset using classical machine learning models to predict late potato blight infection outbreaks before they occur. This predictive approach helps implement preventive measures, significantly reducing the cost and environmental impact of using fungicides post-infection.

Observations for potato blight infection were recorded at 2, 8, 14, and 20 h during the summer. The air humidity varied between 92.58% and 58.74%, and the wind speed ranged from 8 m/s to 20 m/s.

**Figure 2:**
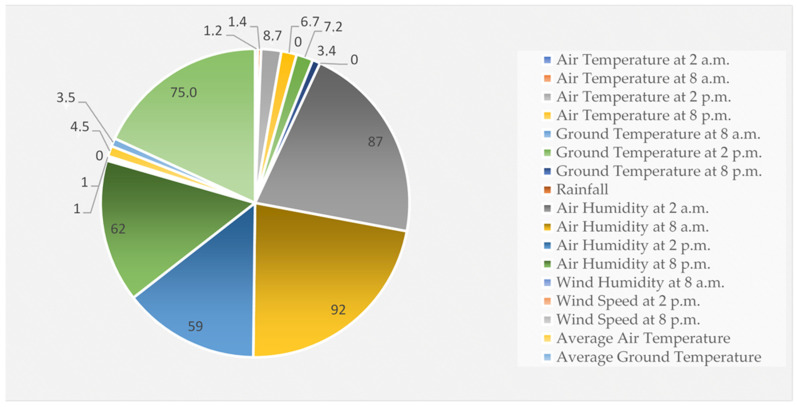
Analysis of meteorological data.

The dataset included meteorological parameters describing the weather conditions during the potato growing season, which are necessary to assess the long-term trends and predict the date of the outbreak of late blight epidemic before it occurs. Table 1 depicts the meteorological conditions for the above specified location. These make it possible to collect data with higher temporal resolution and achieve more accurate modeling and prediction. Improved forecast models based on these data can help farmers make precise decisions about crop protection and epidemic risk management. Figure 2 shows the analysis of the meteorological data.

After analyzing Table 1 of the characteristics of independent variables, we can deepen our interpretation of some of these measures, as follows:

Kurtosis: Values close to zero indicate a near-normal distribution. Negative values for *x*1 (−0.16), *x*3 (−0.19), or *x*6 (−0.26) suggest that the distribution has flatter peaks than the normal distribution. A very high value for *x*8 (26.75) indicates a distribution with a very “pointed” top and long tails.

Skewness: Values close to zero suggest symmetry of the distribution. Negative values, as for *x*2 (0.30), x3 (−0.33), or *x*9 (−2.20), indicate a skew to the left, which means that the longer tail of the distribution is on the left.

Positive values, such as for *x*8 (4.47) or *x*13 (1.34), suggest a tilt to the right, which means a longer tail on the right.

Range: Large range, as for *x*11 (81.00) or x9 (65.00), indicates a wide range of values, which may suggest greater variability and diversity in the data. A small range, as for *x*15 (12.00), suggests that the values of this variable are more concentrated.

Minimum and Maximum: Analyzing minimum and maximum values allows you to understand the extreme values in a dataset. Extremely low minimum values may indicate the presence of outliers or measurement errors.

Additional observations include the following:

Standard Deviation: Standard deviation values, such as for *x*11 (17.66) and *x*12 (14.82), indicate a large variability in the data around the mean. Low standard deviation values, such as for *x*15 (1.75), indicate less variability.

Distribution and Asymmetry: Analyzing kurtosis and skewness together gives a more complete picture of the distribution of data. The variable *x*8, with high kurtosis (26.75) and positive skewness (4.47), indicates a very concentrated distribution with a long right tail.

Central Tendencies: Comparing the mean and median for each variable can help identify the influence of outliers. For example, variable *x*9 (mean 92.58, median 95.00) has a median close to the mean, suggesting a symmetrical distribution with no significant outliers.

Variation Coefficients: These values indicate the relative variability compared to the mean. A high coefficient of variation, as for *x*8 (247.45%) and *x*15 (114.15%), indicates the high variability of the data relative to the mean, which may make predictions difficult. Low values, such as for *x*9 (9.98%) or *x*10 (13.88%), suggests that the data are more stable and predictable.

Variability and Stability: Variability, measured by the coefficient of variation and standard deviation, is crucial when modeling the data because highly changing variables may require special treatment, such as normalization.

Data Distribution: Understanding the distribution of data (skewness and kurtosis) is important when choosing the appropriate analysis and modeling techniques, e.g., whether data transformation is required.

Extreme Values: Identification of outliers (minimum and maximum) may indicate the need for further analysis or data cleaning before applying analytical models.

In summary, descriptive statistical analysis provides valuable information about the structure and characteristics of data, which is crucial for further statistical analysis and predictive modeling.

**Figure 3:**
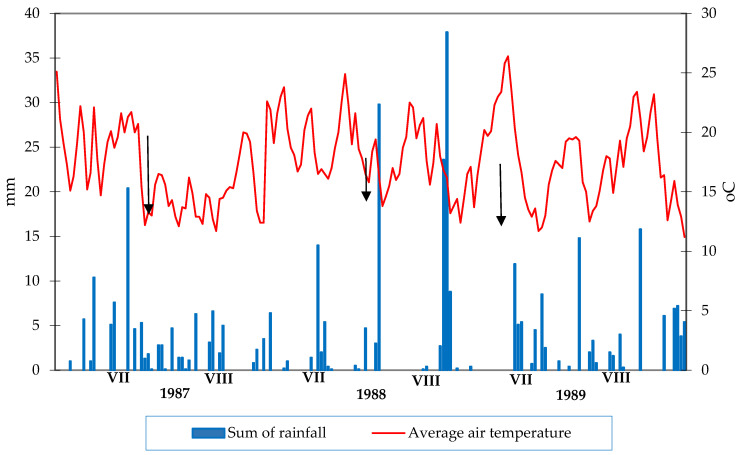
First symptoms of *P. infestans* in the years 1987–1989.

Figure 3 shows the first symptoms (marked by black arrows) of potato blight during the years 1987–1989.

### 3.4. Conditions Favoring Occurrence of Potato Blight

*P. infestans* develops best in specific climatic conditions, which partly occur in Central and Eastern Poland. These include factors like air temperature and humidity, precipitation, sunlight, wind speed, and direction. Optimal temperatures for the development of *P. infestans* range from 15° to 24 °C. High temperatures above 25 °C may inhibit the development of the pathogen; however, nights with temperatures above 10 °C favor the survival and spread of *P. infestans* [24].

High relative air humidity, especially above 90%, is crucial for potato blight. Long periods of wet weather with frequent rainfall and dew create the ideal conditions for the development of the disease.

Precipitation: Frequent rains, especially in summer, may contribute to the spread of potato blight. Rain causes the pathogen’s spores to be transferred to healthy plants. In Central and Eastern Poland, western and southwestern winds predominate, which is the result of the general atmospheric conditions prevailing over Central Europe. Wind direction may vary locally depending on the terrain and local meteorological conditions. In open agricultural areas, wind directions are more consistent with prevailing patterns, while in areas with more forests and hills, there may be greater local variations [24].

Microclimatic conditions: Thickened sowings, limited air circulation, and the microclimate can significantly favor the occurrence and development of *P. infestans*. In Eastern Poland, summers are usually warm and, at the same time, characterized by significant amounts of rainfall and high humidity, which creates favorable conditions for the development of potato blight. Regular monitoring of weather conditions and the use of appropriate plant protection products (such as fungicides, appropriate potato varieties resistant to late blight, and agrotechnical methods) are key to minimizing the risk of a potato late blight epidemic [23].

### 3.5. Data Analysis and Prediction Model

Data on potato blight infection (see Appendix A) include the following: Average infection (y): Assessed on a 9-point scale; Protection (x1): 0 (no protection), 1 (protection); Observation dates (x2): “0” for the first date, “7” for the second date, “14” for the third date, etc.; Nitrogen fertilization (x3): 0, 50, 100, 150, 200 kg N ha^−1^. Infection results were calculated using logarithmic values and statistical formulas for infection rates and variability. Tubers were sampled at harvest and the infection was assessed two months later, with the results statistically analyzed using variation analysis and the Fisher–Snedecor “F” test. The study aims to develop a machine learning model to predict the outbreak of potato blight epidemics based on meteorological data and disease spread rates. Figures and tables illustrate the infection spread rates, regression analyses, and the impact of meteorological conditions on disease development. The ultimate goal is to create a predictive tool to guide farmers in preemptive disease management without the excessive use of fungicides, thereby reducing environmental and economic burden.

### 3.6. Statistics Regarding Potato Blight Infection Data

To estimate the infection of tubers by *P. infestans*, a 10 kg sample of tubers was taken from each combination and each replication plot at the harvest [25]. Two months after the harvest, the infection of tubers by the potato late blight was estimated in all samples. These results were calculated statistically using the variation analysis. The significance of the variability sources was tested by the Fisher–Snedecor “F” test and the significance of differences were tested by Tukey’s test. Because of the large number of samples in which the percentage of infected tubers was close to zero, the statistical analysis was performed using transformed values according to the following:

(1) $$y_{1,2}=arcsin\sqrt{x_{1,2}}\;,$$

where *x_1_* is the percentage of tubers and *x_2_* is the percentage of leaf surface infected by *P. infestans*.

Data describe the potato blight infection for the following four potato varieties: “Beryl”; “Bronka”; “San”; and “Cisa”. There is an extra column where the presence of a flag denotes “protection” and the absence of a flag denotes “no protection”. The statistical results were obtained using linear regression. The infection was expressed as a logarithmic value corresponding with the degrees of the scale [7,8], using the following formula:

(2) $$z_{1,2}=ln\frac{y_{1,2}}{1{-y}_{1,2}}\;,$$

where *y* is expressed in %. This formula allows us to express the percentage of tubers (*y_1_*) and percentage of leaf surface (*y_2_*) infected [26] as a line. The spreading rate of *P. infestans* [27] was defined as a unit increase in the infection over time. For calculation, the dates of the observations were coded in the following way: the first one as “0”; the second one as “7”; the third one as “14”; and so on. The variability of the analyzed results was characterized by the mean arithmetic value calculated from the retransformed values, standard deviation, and coefficient of variability *V*, which was calculated using:

(3) $$V_{1,2}=\frac{S_{1,2}}{{ẑ}_{1,2}}\;100\;,$$

where *S* is the standard deviation and *ẑ* is the mean arithmetic value of a natural logarithm of the final potato blight severity and a natural logarithm of the initial potato blight severity.

The observations of disease development were carried out every seven or ten days, depending on the experimental assumptions. The steps to calculate the rate of potato blight spread are given below.

Collected Data: Information on the area affected by potato blight at different time points was gathered. This involved measuring the size of the affected area in square meters or hectares.

Determined Time Intervals: The time intervals between each data point were determined. For example, measurements were taken weekly, every 10 days, monthly, or at other intervals.

Calculated Change in Area: The change in the affected area for each time interval was calculated. This was performed by subtracting the area affected at the beginning of the interval from the area affected at the end.

Rate of spread: The change in the normalized affected area was divided by the duration of the time interval to calculate the rate of spread. This determined how fast the potato blight was spreading per unit of time (e.g., in a normalized area change per week). The formula to calculate the rate of spread *R* is depicted as follows:

(4) $$R_{1,2}=\frac{{ẑ}_{1,2}}{{t2}_{1,2}-{t1}_{1,2}}\;\;,$$

where *ẑ* is the mean arithmetic value of a natural logarithm of the final potato blight severity and a natural logarithm of the initial potato blight severity; t1 is the number of days from the zero to the appearance of the first symptoms of the disease; and t2 is the number of days from zero to the date when the degree of infection on the next observation date is not greater than the previous one [22,28].

Interpretation: Potato blight spread rates over the time were analyzed to understand how the infection spreads. Patterns such as exponential growth, linear growth, or fluctuations in the rate of spread were observed. It should be remembered that the rate of spread may not remain constant over time and may be influenced by various factors such as meteorological conditions, the presence of late blight-resistant varieties, agricultural practices, etc. Therefore, the results were interpreted considering the potential factors that had influenced the spread of potato blight. Regression equations were calculated each time and were intended to illustrate the rate of spread of potato blight over time in the form of a straight line (these graphs are in Excel). Polish professor Pietkiewicz developed this methodology in the 1970s [23]. After calculating the spread of potato blight, a program was developed based on detailed meteorological data day by day (during the period of the spread of this disease), which allowed us to predict the outbreak of a potato blight epidemic before it occurs.

Potato blight infection is assessed on a reversed 9-point scale, where 9 indicates no signs of disease, and 1 indicates completely infected plants. Therefore, the higher the value, the fewer plants are infected. The methodology and interpretation of the degrees which assess the degree of plant infection by *P. infestans*, are listed as follows [22]:

9—Signifies no symptoms of the disease;
8—Indicates slight symptoms on individual leaves or plant units;
7—Signifies the spread of the disease to several leaves;
6—Indicates moderate plant infection but without apparent symptoms on tubers;
5—Signifies serious leaf infection and the appearance of initial symptoms on tubers;
4—Indicates moderately advanced infection, with visible symptoms on the tubers;
3—Signifies serious infection of both potato leaves and tubers;
2—Indicates severe infection of plants with visible symptoms on leaves and tubers;
1—Signifies destruction of the potato plant.

Meteorological data are crucial here for predicting the onset and course of the potato late blight epidemic. There is a strong correlation between the outbreak of potato blight and the meteorological conditions [29]. Hence, long-term weather data covering a period of 21 years were included in the analysis of the spread rate of the disease. The consequences of potato late blight can be catastrophic, as they lead to yield losses of up to 20–90%, which causes severe losses for farmers.

Many plant protection programs and models predict the development of plant diseases, but none of them can predict potato blight before it occurs. Pesticide companies and large corporations are reluctant to perform such predictions. Nevertheless, our goal is to develop a tool that effectively predicts the onset of an epidemic based on meteorological data and the degree of disease spread. The evaluation of the dataset using classical machine learning models aims to predict the late potato blight infection outbreak before it occurs. By comparing different machine learning models, we identified the classifier that provides the best prediction results.

### 3.7. Description of Potato Blight Infection Data in Period 1987–1989

Regression analyses were performed in a separate spreadsheet (1987–1989), and linear regression coefficients were used to calculate the rate of potato blight spread. Many coefficients were crucial to our analysis. The lines depicting the rate of spread of the disease in the graphs intersected the Y-axis at different points, reflecting the other times when the potato late blight epidemic had occurred. The later the line crossed the y-axis, the longer the vegetative growth of the plants would have been, allowing for higher yields. However, the genetic resistance of potato varieties and meteorological conditions influenced the rate of disease spread. Therefore, weather data are essential for analyzing and predicting *P. infestans* outbreaks. The influence of such meteorological factors as precipitation, air and soil temperature, air humidity, precipitation, sunshine, and wind speed were considered. To effectively predict the development of potato blight, the analysis of meteorological data was combined with the analysis of the speed of the disease spread. This combination enabled us to develop an effective tool to help farmers make informed decisions about protecting their potato crops from this disease. In this worksheet, the rate of spread of potato blight is expressed as a straight line, as shown in Figure 3. When this line intersects with the y-axis, it means 50% of the plants are infected with potato late blight, which means a significant decrease in yields. The goal is to extend this moment as much as possible, extend the growing season, and increase crop accumulation.

Regression analyses were performed in each Excel spreadsheet (1987–1989) and a linear regression coefficient was calculated, which was later used to calculate the rate of spread of potato blight. These lines of disease spread intersected the y-axis at different points (at various times after the first symptoms of potato blight were observed). The later this line intersected the y-axis, the longer the plants would have had vegetative growth, and the higher the yield they would have produced. The graphs show the degree of spread of potato late blight on different varieties, and we can see how resistance to this disease affects the length of the potato and the growing season. However, the weather conditions prevailing in a given year have the greatest impact on the rate of spread of potato late blight and allow us to predict the onset of a late blight epidemic. Our main goal is to compare this rate of disease spread over time with rainfall, temperature, and even wind speed. This can be seen in Figure 3. Figure 3 shows the first symptoms of late blight infection listed in the 1987–1989 datasets.

**Figure 4:**
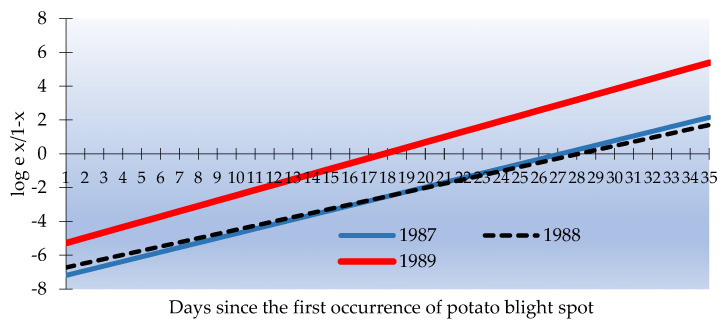
Linear regression model for predicting potato blight infection in period 1987–1989.

We have already discussed the various coefficients (or features) describing metrological data that has been demonstrated in Table 1, based on which we need to deduce a machine learning model. The data related to the potato spread rate can be modeled as a straight line, as shown in Figure 4.

This scale helps farmers and researchers to monitor and assess the level of potato plant infection by taking the appropriate disease control actions, such as using plant protection products, changing cultivation practices, or selecting more resistant potato varieties. The “potato blight spread rate coefficient” refers to the measure used to assess the rate at which the disease spreads within a given area or population of potato plants.

This coefficient helps researchers and agricultural professionals understand disease transmission dynamics and plan the appropriate management strategies to control its spread. Typically, this coefficient is calculated based on various factors such as environmental conditions, host susceptibility, and management practices. A lower coefficient value indicates slower disease spread, while a higher value suggests faster pathogen dissemination within the potato crop. Understanding the potato blight spread rate coefficient is crucial for implementing timely and effective disease management measures, including fungicide applications, crop rotation, and resistant cultivation selection to minimize yield losses and maintain crop health. The intention was to determine the effect of protection against potato late blight by how many days it extends the vegetation of the tested varieties. Potato late blight infection at 50% practically resulted in a quick end to plant vegetation and a yield drop of 20–90%.

**Figure 5:**
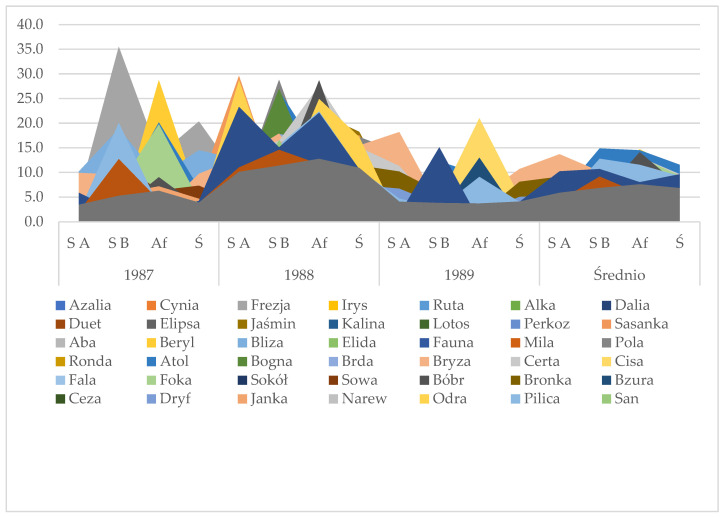
Potato blight infections affecting different varieties of potato over the years 1987–1989. SA—Sencor before emergence; SB—Sencor after emergence in the 10–15 cm phase of potato plants; AF—Afalon 50 WP used before potato emergence as a control plant.

Potato blight [26] could be termed a blight epidemic, and this paper deals with developing a program to predict the outbreak of potato blight because this reduces the potato yield by 30–80% in European countries. Figure 5 shows 44 varieties of potatoes affected by infection over the years 1987–1989. The potato late blight results from all those years (1987–1989) had a different weather pattern, and the date of the outbreak was utterly different in each year. The attached figures present the influence of the application time of the herbicide Sencor 70 WP on the spreading rate of *P. infestans* (Figure 5).

Each value was calculated with a different coefficient of the potato blight spread rate [27]. It is normal that these coefficients differ over the years of research because the weather patterns and conditions of potato blight development would have been different, as can be seen in Figure 5.

### 3.8. Regression Analysis of the Dataset 1987–1989

**Figure 6:**
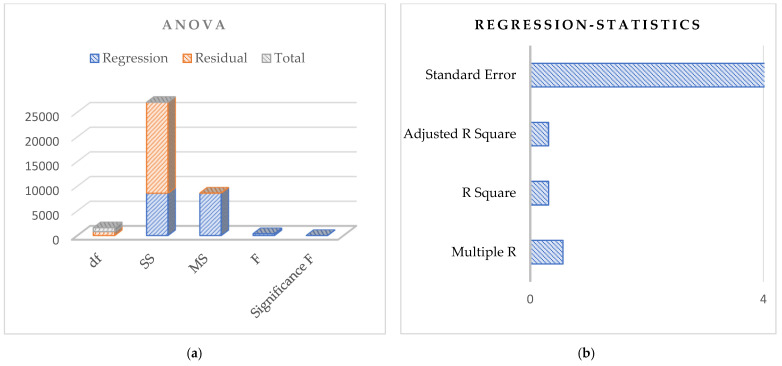
Regression analysis based on potato blight infection data for the year 1987: (**a**) Anova; (**b**) Regression statistics.

**Figure 7:**
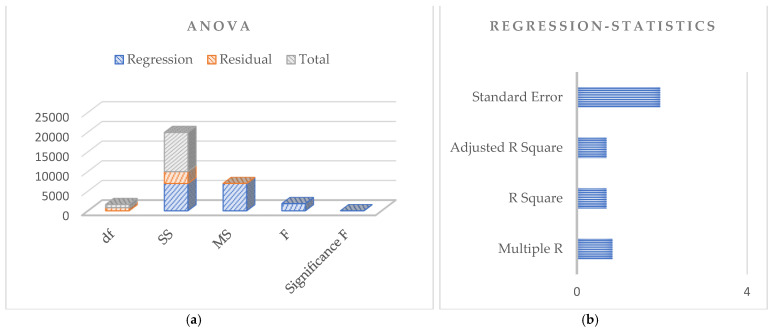
Regression analysis based on potato blight infection data for the year 1988: (**a**) Anova; (**b**) Regression statistics.

**Figure 8:**
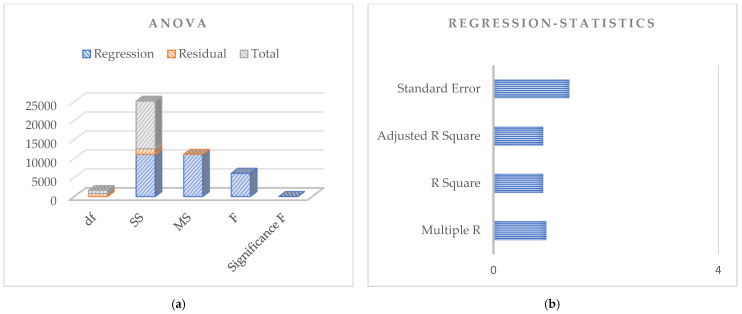
Regression analysis based on potato blight infection data for the year 1989; (**a**) Anova; (**b**) Regression statistics.

Based on the above discussion for the dataset, the regression analysis based on the principles of Pearson’s simple correlation coefficients for the observed data is shown in Figure 6, Figure 7 and Figure 8 for the dependent and independent variables investigated. The figures use 792 observations from the 1987, 1988, and 1989 potato blight infection data. The dependent variable is the rate of spread of potato blight infection, and the independent variable is level of potato blight infection.

Let us first discuss the dataset related to the year 1987, as shown in Figure 6a,b. The standard error obtained here is high, and the correlation among the data is 0.56, depicting a medium correlation between the dependent and the independent variables. In all these tables, the total values, the *p*-value, and the upper confidence limit value at a threshold of 0.95 are shown.

Figure 7a,b present the potato blight infection dataset corresponding to the year 1988. The standard error obtained here is less than the year 1987, and the correlation among the data is 0.83, depicting a high correlation between the dependent and independent variables. These are some reasons why SMOTE analysis was used in our work to generate big data and establish a correlation for efficient model training.

Figure 8a,b show the potato blight infection dataset for the year 1989. The standard error obtained here is less than the year 1988, and the correlation among the data is 0.94, depicting the highest correlation.

### 3.9. Proposed Algorithm

Potato blight appears at different times and circumstances each year as a result of climate change. Therefore, the current dataset contains 21 years of daily meteorological data (climatologists say that these data should cover a period of 20–30 years to be representative) for the potato growing period. The methodology of potato late blight invasion is that, each year, the number of observations changes depending on the date of infection with this pathogen and the weather conditions (in the case of heavy rainfall and high air temperature, potato late blight appears earlier, usually in late May or early June). If it was dry, the plague did not appear until July, but then it would spread very quickly. For each potato variety, the number of observations varied depending on their resistance to *P. infestans*. Varieties resistant to *P. infestans* assimilated longer, and their vegetation was longer than varieties that were less resistant, which would end their vegetation much earlier. Therefore, our main goal was to analyze and predict the rate of spread of potato blight long before its outbreak. The proposed algorithm is subdivided into the following sections:

#### 3.9.1. Machine Learning Methods

Machine learning methods may be broadly classified into the following three main categories:

- Supervised learning;
- Unsupervised learning;
- Semi-supervised learning.

In the case of supervised learning [30] techniques, a target is set along with each class label. The main goal of supervised classification techniques is mapping the input to the output. The different learning techniques under this category are as follows:

(a) Regression: This captures the correlation between the dependent variable and one or more independent variables. There can be different types of regression, e.g., linear regression, non-linear regression, logistic regression, etc.;
(b) Classification: This is used to predict distinct values such as True/False, etc. The different kinds of classification techniques are support vector machines (SVMs), k-nearest neighbors (k-NNs), etc.

Unsupervised learning [31] is another machine learning technique where there are no target values. In this case, the model is trained without target values. The various unsupervised techniques are as follows:

(a) Clustering techniques: K-means clustering, hierarchical clustering, etc.;
(b) Dimensionality Reduction techniques: PCA, ICA, etc.

Semi-supervised learning [32] is a method which falls mid-way between supervised and unsupervised methods. Here, the algorithm is trained on a dataset which may be both labeled and unlabeled, e.g., reinforcement learning.

#### 3.9.2. Data Preprocessing

The next stage is data preprocessing. The first step consists of the collection of meteorological data related to potato blight infection. As discussed in the earlier sections, we have a real-life dataset comprised of historical data along with the corresponding scales of potato blight infection. We also have 18 different meteorological conditions corresponding to potato blight infection as a part of our dataset. In this work, we used the 18 coefficients defined above as inputs, and the potato blight infection as a target variable.

*Feature Selection*: We identified 18 different features, i.e., Airtemp2, Airtemp8, Airtemp81, Airtemp20, Groundtemp8, Groundtemp14, Groundtemp20, Rainfall, AirHumid2, AirHumid8, AirHumid14, AirHumid20, windspeed8, windspeed14, windspeed20, Averageairtemp, Averagegroundtemp, Average_air_Humid.

Our independent variable “x” was already a fusion of all eighteen parameters. Our dependent variable was an “infection” which we wanted to predict based on a fusion of the mentioned eighteen features. So, our 2D matrix of numerals consisted of an “infection” as the target (dependent) variable, and a fusion of all these eighteen features as the independent variable. The rule was to simply concatenate these feature values together in a vector form and then, for multiple levels of infection, we finally had a 2D matrix.

*Data Preprocessing*: In order to increase the dataset, we used an oversampling technique called the SMOTE (Synthetic Minority Oversampling Technique). Our dataset was of size 2773, so it was necessary to oversample the data to increase the number of cases in a balanced way. In order to do this, we developed the following oversampling Algorithm 1:
**Algorithm 1** Smote-AnalysisBegin Smote-Analysis:Step 1: Randomly select two different numbers index1 and index2Step 2: Select a random weight ‘β’Step 3: New_point = β ∗ data[index1] + (1 − β) ∗ data[index2]Step 4: Add the ‘New_point’ to the original data-listEnd

#### 3.9.3. Evaluation Metrics

To evaluate our model, we used the following metrics for evaluation purposes:

Precision: This is the ratio of true positives to total predicted positives;

Recall: This is the ratio of correctly predicted observations to the total sample of observations;

F1-score: This is the harmonic mean of Precision and Recall;

Support: This gives us an idea of the total distribution of a particular class in a dataset;

Confusion matrix: This gives us a pictorial view of the total number of true positives, false positives, true negatives, and false negatives based on which the accuracy is calculated;

Accuracy: This is the ratio of true positives and true negatives to the total number of instances;

Sensitivity: This measures the proportion of actual positives that are correctly identified by the model;

Specificity: This measures the proportion of actual negatives that are correctly identified by the model.

#### 3.9.4. Model Implementation

Using the SMOTE analysis, the number of data samples was increased to 27,000. The next step was the implementation of the model for 5the prediction of the potato late blight infection employing the following classifiers:

- Decision tree classifier;
- Support vector classifier (SVC);
- K-nearest neighbors (KNN) classifier;
- Stacking classifier with logistic regression;
- Stacking classifier with gradient boosting;
- Voting classifier;
- Random forest classifier.

These models handle the non-linear relationships between features and a target variable.

Decision tree classifier: The decision tree classifier was chosen because it could be used both in machine learning and regression analysis. In the present work, we chose 9 levels referring to the 9 different categories of potato blight infection.

Support vector classifier: The SVC can handle non-linearity of data very well, and so we selected this classifier model. In the present work, we selected the “linear” kernel.

KNN classifier: The KNN is a popular supervised learning classifier. This was used here because it could also handle regression as well as classification tasks very well. The test set size chosen was 20% of the training set, and the number of neighbors chosen was 9 different categories of potato blight infection.

Stacking classifier with logistic regression: In order to improve the test accuracy of our dataset, we considered a hybrid classifier. In the present case, we designed a stacking classifier which combined the best performances of the k-nearest neighbors classifier and the random forest classifier.

Stacking classifier with gradient boosting: In this case, in order to obtain better performance, we repeated the previous experiment but now with the gradient boosting classifier. Often, it has been observed that the gradient boosting gives a better prediction than other classifiers because, since it is built on a number of sequential classifiers, its main aim is to correct the errors of the previous classifier. Here, we used the k-nearest neighbors classifier along with the random forest classifier.

Voting classifier: The main purpose of the voting classifier used in the present work was to choose a number of base classifiers and combine them with the predictions of those classifiers to improve the overall performance. In the present work, we used three base classifiers, namely the k-nearest neighbors classifier, the gradient boosting classifier, and the SVC. However, it cannot be guaranteed that the voting classifier will always give the correct result.

Random Forest Classifier: This classifier was used in the present work because it can use a number of decision trees and output a class which is the mode of the classification results of the individual trees. It has also been proven to handle data with high dimensionality.

## 4. Results and Discussion

This section describes the research results obtained, analyzes them, and presents their implications for the research problem. The accuracy rates obtained by each of the above-mentioned classifiers are listed in Table 2 with the different training and test set ratios.

From the results in Table 2, we can confirm that the performance recorded by the decision tree classifier is 78.04%, which is the lowest. The SVC and KNN recorded much higher test accuracies compared to the decision tree classifier, which are 82.30% and 84.02%, respectively.

The stacking classifier with logistic regression (see Appendix B) has given us the maximum rate of potato blight infection detection (87.22%), likely due to the synergy of several well performing models. In the case of the stacking classifier with gradient boosting, being excellent for regression and classification, we achieve a test accuracy of 87.15%. Finally, the performances achieved by the voting classifier and the random forest classifier differ from the earlier hybrid classifiers. We also enlisted the accuracy for different setups of the training and test sets (80:20, 50:50, 70:30, and 90:10) for the original (potato) dataset. The main goal was to reveal if more complicated models would be able to learn better from more data in comparison with less complicated ones. Also, we would be able to see whether it could help us to reveal any overfitting or underfitting models. We found the stacking classifier with logistic regression to be the best performing model, when the split ratio was 90:10.

Table 3 depicts the precision, recall, F1-score, and support for the best-performing classifier (the stacking classifier with logistic regression). The confusion matrix of this classifier is shown in Table 4.

Interpretation of the results is as follows: In the present work, the lowest accuracy of 78.04% rendered by the decision tree classifier suggests that the simplicity of the decision tree model may be insufficient to capture the complexity of the potato blight-related meteorological data. The highest accuracy is achieved using the stacking classifier with logistic regression at 87.22%.

Now, let us analyze the following sensitivity and specificity levels of the stacking classifier with logistic regression in identifying the various levels of potato blight infection rates:

- Class 1: High sensitivity (98%) but lower specificity because many instances from other classes are misclassified into Class 1 (false positives);
- Class 2: Lower sensitivity (37%) indicates that many true instances of Class 2 are misclassified into Class 1, but its specificity is moderate since there are fewer false positives;
- Classes 3, 4, and 7: These have no true positives, leading to 0 sensitivity, but have a perfect specificity since no other class was classified as them.

This report highlights the model’s strong performance in identifying Class 1 but less significant performance with Classes 2, 3, 4, and 7.

Our dataset spans 21 years and includes diverse meteorological conditions, making it unique and comprehensive. The study stands out for utilizing an extensive, real-world meteorological dataset to predict potato blight. Unlike other studies primarily using image datasets, our approach focuses on meteorological data, enabling more comprehensive and realistic predictions. Our dataset and proposed solution represent a significant contribution to this field.

In addition to the above, we also studied feature learning and selection methods [33,34] and listed how the feature selection methods may affect the classification accuracy, which would be helpful for the prediction of potato blight outbreak. We used the following feature selection methods:

(a) Filter Methods: These use statistical techniques (like ANOVA) to evaluate the relationship between the features and the target variables based on their relevance. These methods are very efficient and independent of machine learning models. In this filter method, we used the top 17 features using ANOVA F-statistics.
(b) Wrapper Methods: They evaluate the subset of features by training a machine learning model using the model’s performance as a criterion for selection. Normally, this model gives an optimal performance. Here, we used Recursive Feature Elimination (RFE) with Logistic Regression using *n* = 5 features.
(c) Hybrid Methods: These combine the features of the filter, wrapper, and even embedded methods. Here, dimensionality reduction techniques like PCA are used. The problem with this method is that they can reduce the dimensionality but do not explicitly specify the features. Here, we reduced the dimensionality using logistic regression with a specific threshold.

Table 5 depicts the accuracy of different classifiers after feature learning and selection using a training–test set ratio of 90:10. We see that the decision tree classifier, SVC, and voting and random classifiers, after applying the filter method of feature selection, outperformed methods earlier described in the fourth column of Table 2. However, the wrapper method of feature selection only managed to increase the performance accuracy of SVC compared to the methods earlier described in the fourth column of Table 2. But the hybrid methods performed well only in the case of the SVC, and voting and random forest classifiers. We conclude that feature selection could readily increase accuracies if the features are properly selected. As a part of our future work, we will involve more experiments with feature selection strategies.

Now, let us draw a brief comparison among some of the relevant related works performed in the field of late potato blight infection. Sharma et al. [35] worked on images of potato blight plants and attained an accuracy of 92.9% using the SVM. The significance of this work is how a unified blend of machine learning and image processing can be used to predict late potato blight infection. Our work certainly spans a much larger dataset, but there is presently no imagery in our dataset. Our dataset is also based on real life; however, we still do not have any images in our present dataset. Our work revolves around experimenting with the two-dimensional data available to us but, in the future, an integration of 2D data with real-life images of potato blight infection will be our next research priority.

In another interesting work [36], Iqbal et al. investigated 450 images of healthy and diseased potato leaves taken from early blight, late blight, and healthy/non-diseased potato leaf datasets, and they applied a hybrid system comprising machine learning and image processing. Although the dataset used was small in size, the authors used seven classifiers, and the highest accuracy of 97% was rendered by Random Forest Classifier.

Anim-Ayeko et al. [37] also worked on a method for detecting early and late blight in potatoes and tomatoes. The authors proposed a ResNet-9 model to detect blight diseases of potatoes. The authors attained a test accuracy of 99.25% using the Resnet-9 model, but that was also using the dataset comprised of images of potato blight infection.

Patil et al. [38] worked on detecting potato leaf image diseases by integrating CNN with LSTM. The model was analyzed on the basis of accuracy, precision, recall, and F1-score. The proposed model attained an accuracy of 99.02% and outperformed the CNN, LSTM, and MobileNet models.

Potato blight infection, which has been addressed in the above works, was not examined based on a real-time dataset. If one has to efficiently evaluate the rate of spread of potato blight infection, the exact ground level meteorological conditions need to be collected, otherwise the prediction will be incorrect. True to our knowledge, there is currently no work which has addressed how to detect late potato blight infection *(P. infestans*) [35] long before their outbreak using machine learning techniques. The introduction of this dataset and solution to this epidemic is our primary contribution. We have introduced a real-life meteorological dataset, along with the 18 coefficients for weather conditions, from 1980 to 2000. The potato blight infection data for the years 1987–1989 have also been given.

## 5. Limitations and Future Work

Potato late blight is a serious infection affecting potato plants in major parts of Europe. This can lead to further economic degradation because, till today, potatoes are a staple food consumed in all forms and by many people, not only in Europe but in many parts of the world. Although there have been many studies conducted in this field, there are still plenty of research gaps which need to be addressed. Potato blight is a serious infection which diminishes the economic yield of any country from 20% to 90%.

There are many factors which contribute to late blight infection. First would be the weather conditions, as can be seen from the prevailing temperatures in parts of Europe and Poland, where the potato blight infection was initially detected. The temperatures (highest and lowest) are also different at different times of the year. Other factors that could aggravate the spread of potato blight infection include ground temperature and wind speed. There are other factors, such as average and maximum soil water [9]. The above factors have a strong correlation with the rate of spread of potato blight infection.

Keeping the above factors in mind, we have proposed a 21-year dataset which summarizes the various meteorological conditions that can affect the spread rate of potato blight infection.

In order to solve the problem of prediction, logistic regression is an effective tool, as demonstrated in this paper. The significance level and the explanatory variables associated with regression help us to analyze the level of correlation between the climatic conditions and how they are related to the rate of spread of potato blight infection [39,40]. Regression can help us detect changes in the rate of potato blight infection by varying the levels of various meteorological conditions and minimizing the error involved in doing so. However, one of the key limitations of regression analysis is that it cannot predict the rate of spread of potato blight beforehand. If machine learning techniques are incorporated to detect the rate of spread of potato blight infection [41] beforehand, then the use of costly fertilizers can be reduced.

On the basis of the extensive experimentation conducted in this paper, we can conclude that the current approach could be more effective by incorporating additional variables or data from other sources. This may involve additional climate parameters, soil fertility conditions, and some visual aspects of the potato blight infection data.

Also, testing on even larger datasets could confirm the model’s effectiveness. The size of our dataset is not too large to showcase greater variability. In future, the size of the original dataset needs to be bigger.

## 6. Conclusions

Our study focuses on mitigating late potato blight infection through a machine learning model using supervised classifiers. This model predicts the occurrence of late blight based on meteorological data, enabling preemptive measures to control the disease. Our results demonstrate that machine learning techniques can predict potato blight early, reducing the reliance on costly fertilizers and fungicides.

Key findings include the following:

- Machine learning models, particularly the stacking classifier with logistic regression, achieved high detection rates;
- Predictive accuracy can be enhanced using cross-validation and accumulating diverse datasets;
- Climate change significantly affects potato blight spread and severity, making the adaptive strategies essential.

While deep learning is powerful, sometimes, it is not the best choice because of the following:

- Traditional models can perform better with smaller datasets;
- Traditional models require less computational power and are faster;
- Models such as decision tree and linear regression offer more interpretability compared to deep learning.

Deep learning is suitable for temperature prediction tasks, especially when dealing with complex spatial and temporal patterns. For example, the growth of plants is not only affected by various temperature and soil conditions but also other plants/species which are growing nearby. Complex spatial patterns result in situations which are affected by multiple factors. Our dataset is not so complex because, here, we have eighteen features like air and ground temperature, wind direction and speed, humidity, rainfall, etc. Traditional supervised learning models are generally more appropriate and likely to yield a better performance, which has been shown in the “Experimental Results” section. At present, we do not know how these features will affect the final potato infection, so by focusing on traditional supervised learning models, we can achieve good predictions for potato blight infection, leveraging the strengths of these methods for structured numerical data. Future work will involve testing the model on unseen data, applying cross-validation techniques, and exploring deep learning models to further improve prediction accuracy.

## Figures and Tables

**Table 1:** Descriptive statistics of meteorological data (independent variable x).

Specification	*x1*	*x2*	*x3*	*x4*	*x5*	*x6*	*x7*	*x8*	*x9*	*x10*	*x11*	*x12*	*x13*	*x14*	*x15*	*x16*	*x17*	*x18*
Mean	10.82	13.42	18.35	15.14	13.66	19.75	16.00	1.90	92.58	83.30	58.74	74.78	2.36	3.54	1.53	14.43	16.47	77.35
Standard error	0.11	0.11	0.12	0.11	0.11	0.13	0.13	0.09	0.18	0.22	0.34	0.28	0.04	0.05	0.03	0.10	0.11	0.19
Median	11.00	14.20	18.80	15.80	14.75	20.20	17.20	0.00	95.00	85.00	56.00	75.50	2.00	3.00	1.00	15.13	17.42	77.75
Standard deviation	6.00	5.97	6.52	5.87	6.01	6.74	6.99	4.71	9.24	11.56	17.66	14.82	2.01	2.41	1.75	5.18	6.04	10.26
Kurtosis	−0.16	0.19	−0.19	−0.21	0.12	−0.26	−0.36	26.75	5.41	−0.17	−0.63	−0.71	3.16	1.21	4.86	−0.10	−0.42	−0.07
Skewness	0.01	−0.30	−0.33	−0.47	−0.29	−0.33	−0.54	4.47	−2.20	−0.62	0.45	−0.29	1.34	0.96	1.94	−0.47	−0.44	−0.31
Range	30.70	39.00	38.60	31.10	34.20	36.60	30.70	52.00	65.00	60.00	81.00	71.00	17.00	17.00	12.00	30.70	30.13	66.50
Minimum	−4.70	−4.20	−3.20	−3.00	−0.40	0.10	−0.10	0.00	35.00	40.00	19.00	29.00	0.00	0.00	0.00	−2.85	−0.10	33.00
Maximum	26.00	34.80	35.40	28.10	33.80	36.70	30.60	52.00	100.00	100.00	100.00	100.00	17.00	17.00	12.00	27.85	30.03	99.50
Variation coefficients (%)	55.45	44.47	35.54	38.80	44.00	34.14	43.72	247.45	9.98	13.88	30.07	19.82	85.10	68.05	114.15	35.92	36.65	13.26

*x1*—Air Temperature at 2 a.m. in °C; *x2*—Air Temperature at 8 a.m. in °C; *x3*—Air Temperature at 2 p.m. in °C; *x4*—Air Temperature at 8 p.m. in °C; *x5*—Ground Temperature at 8 a.m. in °C; *x6*—Ground Temperature at 2 p.m. in °C; *x7*—Ground Temperature at 8 p.m. in °C; *x8*—Rainfall in mm; *x9*—Air Humidity at 2 a.m. in %; *x10*—Air Humidity at 8 a.m. in %; *x11*—Air Humidity at 2 p.m. in %; *x12*—Air Humidity at 8 p.m. in %; *x13*—Wind Speed at 8 a.m. in m/sec; *x14*—Wind Speed at 2 p.m. in m/sec; *x15*—Wind Speed at 8 p.m. in m/sec; *x16*—Average Air Temperature in °C; *x17*—Average Ground Temperature in °C; *x18*—Average Air Humidity in %.

**Table 2:** Accuracy of different classifiers.

Classifier Used	Train/Test Set Ratio			
	80:20	50:50	70:30	90:10
Decision Tree Classifier	78.04	76.56%	77.68%	78.56%
SVC (Support Vector Classifier)	82.30	81.43%	82.25%	80:59%
KNN Classifier	84.02	83.73%	84.36%	85.41%
SC + Logistic Regression	87.22	86.87%	87.89%	88.63%
SC + Gradient Boosting	87.15	86.83%	87.81%	88.26%
Voting Classifier	85.28	84.28%	85.21%	86.04%
Random Forest Classifier	85.96	85.37%	86.06%	86.56%

**Table 3:** Precision, recall, F1-score, and support for stacking classifier with logistic regression.

Classification	Precision	Recall	F1-Score	Support	Sensitivity	Specificity
1	0.88	0.98	0.93	4448	0.97	0.37
2	0.80	0.37	0.51	947	0.37	0.97
3	0.00	0.00	0.00	1	0	1.0
4	0.00	0.00	0.00	2	0	1.0
7	0.00	0.00	0.00	2	0	1.0
Approximate accuracy	87%					
macro avg	0.33	0.27	0.29	5400		
weighted avg	0.86	0.87	0.85	5400		

**Table 4:** Confusion matrix for stacking classifier with logistic regression.

	**PREDICTED CLASS**					
**TRUE CLASS**		**1**	**2**	**3**	4	7
	1	4357	91	0	0	0
	2	594	393	0	0	0
	3	1	0	0	0	0
	4	2	0	0	0	0
	7	2	0	0	0	0

**Table 5:** Accuracy of different classifiers after feature learning and selection.

Classifier Used	Filter Methods	Wrapper Methods	Hybrid Methods
Decision Tree Classifier	79.41%	73.89%	76.89%
SVC (Support Vector Classifier)	83.67%	82.56%	82.56%
KNN Classifier	84.63%	83.04%	85.44%
Stacking Classifier + Logistic Regression	88.48%	84.26%	87.52%
Stacking Classifier + Gradient Boosting	88.11%	84.00%	87.15%
Voting Classifier	86.22%	83.63%	86.44%
Random Forest Classifier	86.74%	83.85%	86.85%

## Data Availability

The raw data in this article will be made available by the authors on request.

## Abbreviations

CRISPR	Clustered Regularly Interspaced Short Palindromic Repeats
*P. infestans*	Phytophthora infestans
ML	Machine Learning
AI	Artificial Intelligence
ARIMA	Autoregressive Integrated Moving Average with exogenous variables
NNAR	Neural Network Auto Regression
SVR	Support Vector Regression
CART	Classification and Regression Tree
SA	Sencor before emergence
SB	Sencor after emergence in the 10–15 cm phase of potato plants
SMOTE	Synthetic Minority Oversampling Technique
AF	Afalon 50 WP used before potato emergence as a control plant
SVM	Support Vector Machine
SVC	Support Vector Classifier
KNN	K Nearest Neighbor
RF	Random Forest

## Appendix A

Potato blight infection (commonly referred to as “potato blight”) is assessed on a reversed 9-point scale, where 9 indicates no signs of disease and 1 indicates completely infected plants. Therefore, the higher the value, the fewer plants are infected. However, in the context of the formulas used, it seems that potato blight infection is inversely proportional to the values expressed in these formulas. This means that the higher the value of meteorological parameters (humidity and temperature), the lower the value of infection expressed in these formulas:

−7470 + 0.315 ∗ C6,

−6.978 + 0.288 ∗ F6,

−5.602 + 0.314 ∗ I6.

These formulas do not consider any meteorological conditions; they are only used to calculate the rate of potato blight spread. In this work, we considered the meteorological conditions in order to predict the rate of spread of potato blight infection.
